# A Rare Case of Grey Zone Lymphoma Successfully Treated with Brentuximab Vedotin and R-CHP Chemotherapy

**DOI:** 10.1155/2019/4121234

**Published:** 2019-04-11

**Authors:** Rajiv M. Mallipudi, Lance Alquran, Vishnu A. Shenoy, Lori A. Leslie, John A. Conti

**Affiliations:** ^1^Hackensack Meridian Health Mountainside Medical Center, Department of Internal Medicine, 1 Bay Ave, Montclair, NJ 07042, USA; ^2^University of Delaware, Newark, DE 19716, USA; ^3^Hackensack Meridian Health Hackensack University Medical Center, John Theurer Cancer Center, 92 2nd Street, Hackensack, NJ 07601, USA

## Abstract

**Introduction:**

The diagnosis of B-cell lymphoma, unclassifiable, with features intermediate between diffuse large B-cell lymphoma (DLBCL) and classical Hodgkin's lymphoma (cHL), also referred to as grey zone lymphoma (GZL), is a challenging diagnosis. There are no standardized guidelines; however, evidence strongly suggests that DLBCL-based regimens are effective in the treatment of GZL. Brentuximab vedotin (BV) is an anti-CD30 antibody drug conjugate that has established efficacy in relapsed/refractory Hodgkin and some T-cell lymphomas. There is some evidence that BV has a positive response in non-Hodgkin lymphoma (NHL) with a wide range of CD30 expressions—including GZL.

**Case:**

We present a case of a patient initially diagnosed with cHL who underwent repeat biopsy which was revealed to be GZL. Based on PET scanning and immunohistochemical studies, she was classified as a stage IIIA CD20+/CD30+ GZL patient. Given her strong CD30 expression, she underwent 6 cycles of R-BV-CHP (rituximab, brentuximab vedotin, cyclophosphamide, doxorubicin, and prednisone) chemotherapy and achieved complete response (CR) both clinically and radiographically.

**Discussion:**

Given the rarity of GZL, this case illustrates the immense challenges in making the diagnosis, discusses the current treatment options, and suggests that BV may be a viable therapeutic candidate in the treatment of GZL.

## 1. Introduction

GZL is a rare lymphoma that presents with a wide spectrum of morphologies and immunohistochemical feature characteristics of both cHL and DLBCL in the same tumor tissue sample, thus necessitating extensive sampling to make the correct diagnosis [1–3]. GZL often presents at an early stage with B symptoms and mediastinal mass in males aged 20-40 years [1, 2, 4, 5]. Given the rarity of the condition and lack of clinicopathological prognostication, the management of GZL is particularly challenging as there is no standard of care [1, 5–7]. At this time, DLBCL-based regimens are effective in the treatment of GZL compared to cHL treatments [1, 6, 8]. Brentuximab vedotin (BV) is an anti-CD30 antibody drug conjugate that has established efficacy in relapsed/refractory Hodgkin and some T-cell lymphomas. There is some evidence that BV has a positive response in NHL with a wide range of CD30 expressions—including GZL. We present a case of GZL that was initially diagnosed as cHL and then successfully treated with 6 cycles of BV with R-CHP (rituximab, cyclophosphamide, doxorubicin, and prednisone).

## 2. Case Presentation

A 77-year-old woman with prediabetes presented to her primary care doctor for a routine preventative care visit with a new presentation of left-sided axillary lymphadenopathy. She had a screening mammogram performed and a breast ultrasound that showed a 5 mm left breast nodule and left axillary adenopathy up to 3.2 cm. Given concern for breast cancer, she underwent a core needle biopsy of the left axillary node and was diagnosed with classical Hodgkin's lymphoma (cHL) with immunostaining showing large atypical cells that were CD30+, CD15+ (subset), CD20+ (strong), and PAX5+. No flow cytometric analysis was performed. The local pathologist read the specimen as most likely representing a cHL, although the pathologist considered alternatively that the specimen could represent non-Hodgkin lymphoma (NHL) of the diffuse large B-cell lymphoma (DLBCL) subtype.

She was referred to a medical oncologist with no B symptoms and an unremarkable physical exam except for diminished hearing in the left ear and left axillary adenopathy. Her labs were notable for hemoglobin 11.9 g/dL, leukocytes 7,600/*μ*L, absolute neutrophil count 5,400/*μ*L, absolute lymphocyte count 1,500/*μ*L, platelets 261,000/*μ*L, albumin 4.6 g/dL, and LDH 198 U/L (upper limit of normal 243 U/L). She underwent staging with a PET/CT scan and bone marrow biopsy of the right posterior superior iliac crest. The bone marrow morphology showed normocellular marrow (30%) with maturing trilineage hematopoiesis and no evidence of cHL. The flow cytometric analysis also showed no evidence of a B- or T-cell lymphoproliferative disorder. The PET/CT scan from the skull base to the midthigh revealed hypermetabolic adenopathy within the neck, chest, abdomen, and pelvis with the largest area of bulky adenopathy in the left axilla (largest measuring 3.3 × 1.7 cm with SUV 14.5) and a left subpectoral adenopathy (measuring 2.9 × 1.0 cm with SUV 12.3). There was no focal hypermetabolic activity within the liver or spleen. However, there was involvement of a few left paraaortic retroperitoneal lymph nodes and inguinal lymph nodes below the diaphragm. She was therefore staged as stage IIIA.

The patient was sent for a second opinion at a tertiary care referral center to confirm the diagnosis and advised on treatment options. The second opinion oncologist recommended excisional biopsy of the left axillary node for diagnostic clarity. This lymph node biopsy was read by the local pathologist as cHL against a background of extensive nonnecrotizing granulomatous inflammation. The H&E sections demonstrated a lymph node with effaced architecture and nonnecrotizing granulomatous inflammation and scattered large atypical binucleated cells with prominent central nuclei, reminiscent of Reed-Sternberg cells seen in cHL. The background was lymphoplasmacytic with histiocytic infiltrate. On the immunohistochemical studies, the large atypical cells were CD15+, CD30+, CD20+ (bright), PAX5+, MUM1+ and CD3-, CD10-, CD45-, and BCL2-. The flow cytometric analysis showed no immunophenotypic evidence of a B-cell or T-cell lymphoproliferative disorder.

**Figure 1:**
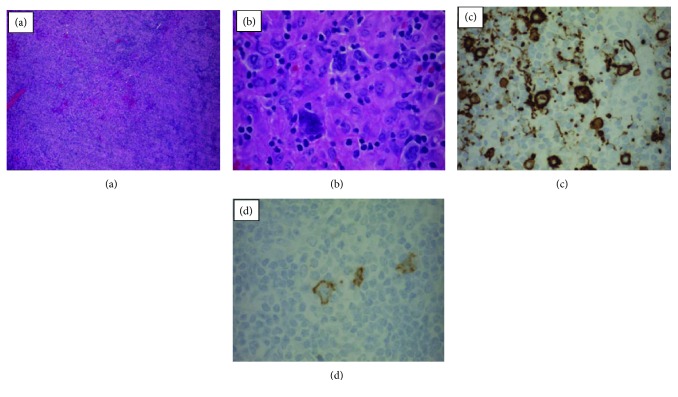
The tissue from the left axillary lymph node biopsy revealed architectural and cytological features consistent with a diagnosis of grey zone lymphoma (GZL). (a) Effacement of the nodal architecture by scattered large and atypical lymphoid cells with abundant amounts of pale cytoplasm, irregular nuclear contours, vesicular chromatin, and prominent nucleoli. (b) Frequent binucleated and multinucleated forms are also seen. Intermixed are large sheets of histiocytes with abundant cytoplasm. The tissue includes a significant number of neoplastic cells within the stromal background and includes the broad cytological spectrum with marked nuclear pleomorphism and shows strong presence of atypical lymphocytes. Immunohistochemical stains show that these atypical lymphocyte tumor cells are strongly positive for CD20+ (c) and positive for CD30+ (d).

However, a second opinion by the pathology group at the tertiary care referral center rendered a diagnosis of B-cell lymphoma, unclassifiable, with features intermediate between DLBCL and cHL, also referred to as grey zone lymphoma (GZL) [9]. While the morphology of the atypical lymphocytes and presence of CD30+ expression were suggestive of cHL, the strong expression of multiple B-cell markers (CD20+, PAX5+, and OCT2+) was not typical (Figure 1). Furthermore, in situ hybridization EBV mRNA (EBER-ISH) was positive in many large cells. This case was discussed at an interdisciplinary conference at the tertiary referral care center, and a final diagnosis of GZL was made. Prior to beginning an anthracycline-based treatment regimen, she underwent cardiac echocardiography which revealed a normal ejection fraction of 60-65% with no valvular disease.

She remained at the referral center for treatment of her stage IIIA CD20+/CD30+ GZL and underwent 6 cycles of R-BV-CHP (rituximab, brentuximab vedotin, cyclophosphamide, doxorubicin, and prednisone) therapy not as part of a clinical trial, with restaging PET scan after cycle 3 which showed complete response (CR). After completing the total course of 6 cycles of R-BV-CHP, she underwent an end-of-treatment PET scan consistent with CR. She had no significant side effects from her treatment. Since June 2018, the patient remains in complete remission and retains an excellent performance status.

## 3. Discussion

GZL can present with a wide spectrum of morphologies with features of both cHL and DLBCL in the same tumor tissue sample, thus necessitating extensive sampling to make the correct diagnosis [1–3]. In fact, the initial misdiagnosis of cHL was based on a core needle biopsy, which is inappropriate to diagnose GZL [10]. Typically, the diagnosis of GZL is obtained by a more invasive excisional or incisional biopsy that requires expert pathologic evaluation of the involved tissue [10]. It was only after an excisional biopsy was done for this patient that the diagnosis of GZL was successfully made. A key feature of GZL is the abundance of tumor cells, typically as a confluent sheet in a background containing a paucity of inflammatory cells; however, eosinophils, histiocytes, and small lymphocytes are seen [2]. GZL can contain a variable level of fibrosis, and the neoplastic nuclei have a greater range in size and shape, with more infrequent eosinophilic nucleoli than the Reed-Sternberg cells seen in cHL disease [2, 3]. Similar to morphology, GZL immunophenotypically has transitional features between cHL and DLBCL. The most prevalent immunohistochemistry (IHC) in GZL is CD20+, CD30+, MUM1+, CD79+, PAX5+, and Oct2+ [2–4, 6]. CD30 is a cell membrane protein detectable by IHC in about 8% of primary mediastinal large B-cell, 20% of diffuse large B-cell (DLBCL), and 100% of GZL [11].

Typically, stage III cHL is treated with ABVD (doxorubicin, bleomycin, vinblastine, and dacarbazine) which has been the standard regimen for several decades [12]. Alternative approaches to stage III cHL described by the National Comprehensive Cancer Network (NCCN) also include Stanford V (doxorubicin, vinblastine, mechlorethamine, vincristine, bleomycin, etoposide, and prednisone) with involved-site radiation therapy (ISRT) in selected patients and escalated BEACOPP (bleomycin, etoposide, doxorubicin, cyclophosphamide, vincristine, procarbazine, and prednisone) with ISRT in selected patients [12]. Recently, the FDA approved the use of brentuximab vedotin (BV) combined with AVD (doxorubicin, vinblastine, and dacarbazine) treatment as a potential treatment option for stage III and IV cHL [12].

Alternatively, stage III DLBCL-like lymphomas are typically treated with R-CHOP (rituximab, cyclophosphamide, doxorubicin, vincristine, and prednisone), DA-EPOCH-R (dose-adjusted etoposide, prednisone, vincristine, cyclophosphamide, doxorubicin, and rituximab), or more intensive anthracycline-based regimens for certain high-risk subtypes [6].

Given the rarity of the condition and lack of clinicopathological prognostication, the management of GZL is particularly challenging as there is no standard of care [1, 5–7]. Recent studies suggest that treatment of GZL with DLBCL-based regimens is effective, including the use of R-CHOP as well as DA-EPOCH-R [1, 6, 8].

BV is an anti-CD30 antibody drug conjugate (ADC) that has established efficacy in relapsed/refractory Hodgkin and some T-cell lymphomas [11, 13–15]. A recent review paper also showed that BV had a positive response in NHL with a wide range of CD30 expressions [15, 16]. CD30 positivity is seen in GZL cases; BV is an attractive targeted approach. While typically vincristine (Oncovin) is used in the standard R-CHOP regimen for DLBCL, it was replaced with BV in this patient since her GZL strongly expressed CD30 positivity. Due to overlapping toxicity of peripheral neuropathy and similar mechanisms of action as antimicrotubule agents, vincristine was replaced by BV rather than simply adding BV to CHOP regimen.

A recent trial explored the use of BV with R-CHP (rituximab, cyclophosphamide, doxorubicin, and prednisone) as frontline treatment for CD30+ primary mediastinal large B-cell, DLBCL, and GZL [11]. The R-BV-CHP regimen demonstrated activity in all three lymphoma types. There were two GZL patients in the trial; one remains in CR after the chemotherapy followed by an autologous stem cell transplant. Regimens incorporating BV in the management of GZL show promise and are being further explored in ongoing clinical trials [11, 16, 17].

Our patient had stage IIIA GZL with strong CD30+ expression, received 6 cycles of R-BV-CHP chemotherapy, and achieved CR both clinically and radiographically. She has remained in complete remission since the end of her treatment in June 2018.

Based on the initial diagnosis of cHL, this patient would have been treated with an ABVD-based regimen, rather than a DLBCL-based regimen which was the preferred approach in this case of GZL. Fortunately, through interdisciplinary collaboration between medical oncology and pathology at the tertiary care referral center, the patient was able to be diagnosed and subsequently properly managed for the rare condition of grey zone lymphoma.
